# The Firegoose: two-way integration of diverse data from different bioinformatics web resources with desktop applications

**DOI:** 10.1186/1471-2105-8-456

**Published:** 2007-11-19

**Authors:** J Christopher Bare, Paul T Shannon, Amy K Schmid, Nitin S Baliga

**Affiliations:** 1 Institute for Systems Biology, 1441 N 34th Street, Seattle, WA 98103, USA

## Abstract

**Background:**

Information resources on the World Wide Web play an indispensable role in modern biology. But integrating data from multiple sources is often encumbered by the need to reformat data files, convert between naming systems, or perform ongoing maintenance of local copies of public databases. Opportunities for new ways of combining and re-using data are arising as a result of the increasing use of web protocols to transmit structured data.

**Results:**

The Firegoose, an extension to the Mozilla Firefox web browser, enables data transfer between web sites and desktop tools. As a component of the Gaggle integration framework, Firegoose can also exchange data with Cytoscape, the R statistical package, Multiexperiment Viewer (MeV), and several other popular desktop software tools. Firegoose adds the capability to easily use local data to query KEGG, EMBL STRING, DAVID, and other widely-used bioinformatics web sites. Query results from these web sites can be transferred to desktop tools for further analysis with a few clicks.

Firegoose acquires data from the web by screen scraping, microformats, embedded XML, or web services. We define a microformat, which allows structured information compatible with the Gaggle to be embedded in HTML documents.

We demonstrate the capabilities of this software by performing an analysis of the genes activated in the microbe *Halobacterium salinarum NRC-1 *in response to anaerobic environments. Starting with microarray data, we explore functions of differentially expressed genes by combining data from several public web resources and construct an integrated view of the cellular processes involved.

**Conclusion:**

The Firegoose incorporates Mozilla Firefox into the Gaggle environment and enables interactive sharing of data between diverse web resources and desktop software tools without maintaining local copies. Additional web sites can be incorporated easily into the framework using the scripting platform of the Firefox browser. Performing data integration in the browser allows the excellent search and navigation capabilities of the browser to be used in combination with powerful desktop tools.

## Background

Information resources on the World Wide Web play an indispensable role in modern biology. Using a web browser, the biologist can easily access a wealth of information such as sequences, biochemical pathways, protein interactions, functional domains, annotations, and gene expression data. Yet, integrating data from diverse sources remains challenging. Biologists wishing to analyze their own experimental data in combination with publicly available data face the cumbersome tasks of converting file formats, reconciling incompatible schemas, and mapping between inconsistent naming systems. Several strategies for data integration have been developed in the context of biology [1]. Data warehousing and web services are two general approaches. Semantic mapping between different data sources has been accomplished by describing biological entities in terms of shared object models [2] or curated ontologies [3]. Our team's previous work in this area resulted in the Gaggle, a software environment that integrates databases and software tools based on a few simple data types and the principle of semantic flexibility [4]. Previously, the Gaggle supported integration with web resources through a special purpose browser of limited capabilities. An improved solution was needed due to the ubiquitous role of the browser and the increasing relevance of the web to data integration.

The web is rapidly becoming a channel for structured data as evidenced by the increasing adoption of web services. Rich internet applications, microformats [5], and the Semantic Web [6] are pushing the browser into a central role as an information broker [7] working with data in human and machine readable form side-by-side. Several tools outside the domain of biology have augmented the browser with these types of capabilities, including Greasemonkey [8], Piggy Bank [9], and Operator [10]. These technologies offer the ability to perform ad-hoc manipulation of data from multiple sources on the web using familiar browser based interfaces.

We developed the Firegoose to bring a full-featured browser into the Gaggle environment and to allow easy transfer of data between the web and the desktop. The Firegoose is a toolbar for the Mozilla Firefox [11] browser that makes use of a diverse array of web communication protocols to seamlessly query and retrieve data from custom databases as well as popular bioinformatics resources including KEGG [12], EMBL STRING [13,14], and DAVID [15]. As a member of the Gaggle software environment, the Firegoose can also exchange data with a growing collection of desktop tools. Bringing the data integration techniques of the Gaggle into the browser, the Firegoose facilitates exploratory analysis of systems biology data using web resources and desktop software tools.

## Implementation

The Firegoose is implemented as an add-on extension to Mozilla Firefox, a popular opensource web browser. Its user interface is a toolbar defined in XUL, an XML dialect for describing user interface components, and its actions are scripted in Javascript. The Firegoose communicates with the Gaggle via the Java RMI protocol and supports several modes of information exchange over the web.

### The Gaggle approach

While it can be useful outside of the Gaggle, the Firegoose is a component of the Gaggle software environment. The goal of the Gaggle is to enable interactive exploration of interrelated biological data by making data transfer between analysis tools, databases, and now web resources, quick and easy. Integration is achieved by supporting the interchange of five simple data types, which cover a wide variety of use-cases within the domain of systems biology.

**Name list**: a list of identifiers.

**Associative array**: a set of key/value pairs.

**Data matrix**: a 2 dimensional grid of floating point numbers, with labels for each row and column.

**Network**: a graph in which attributes may be assigned to nodes and edges.

**Cluster**: a set of identifiers of interest under a set of conditions.

The central feature of the Gaggle is the *broadcast*. Applications become part of the Gaggle by implementing an interface that allows them to accept broadcasts. A broadcast consists of sending a message holding one of the five data types to a program called the Gaggle Boss, which then relays the message to one or more receiving applications or targets.

Using the simple Gaggle data types as an intermediary has the advantage that applications can share data without sharing common object models. Each program is free to handle broadcasts in a way that makes sense in its own context. This is *semantic flexibility*, the first of the Gaggle's guiding principles. The second is to keep the set of data types as small as possible minimizing the programming effort required to integrate a new application into the Gaggle environment.

The Gaggle environment currently includes Cytoscape [16] for viewing networks, the R statistical package [17], Multi-experiment Viewer (MeV) [18] for microarray analysis, the spreadsheet-like Data Matrix Viewer (DMV), and several other programs. The term "gaggled" is used to indicate that a software application or database has been configured to exchange messages with the Boss; for example, Cytoscape has been gaggled through the Cygoose plug-in.

**Figure 1:**
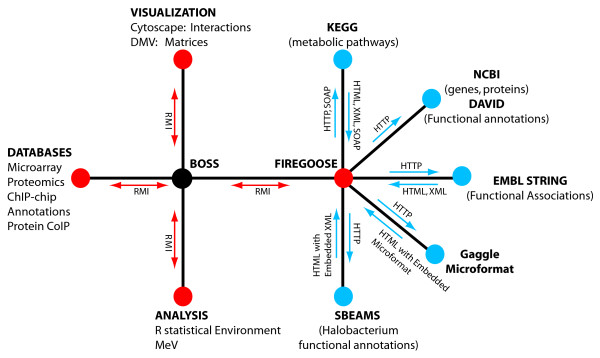
**Communication in the Gaggle**. Software and databases shown as red dots send and receive broadcasts via Java RMI. The blue nodes are web resources connected to the Gaggle through the Firegoose and accessed using HTTP with other protocols and formats such as HTML, XML, and SOAP layered over top.

The Firegoose uses the power of the browser to extend the reach of the Gaggle to target web resources based on widely supported protocols. The Firegoose communicates with the Gaggle Boss via Java RMI. To communicate with web resources, the Firegoose supports standard web protocols and data encoding schemes such as HTTP, HTML, XML, and SOAP. A schematic view (Fig. 1) shows the flow of messages over various protocols between desktop applications, the Gaggle Boss, the Firegoose, and several websites.

### Exchanging data with web resources

**Figure 2:**
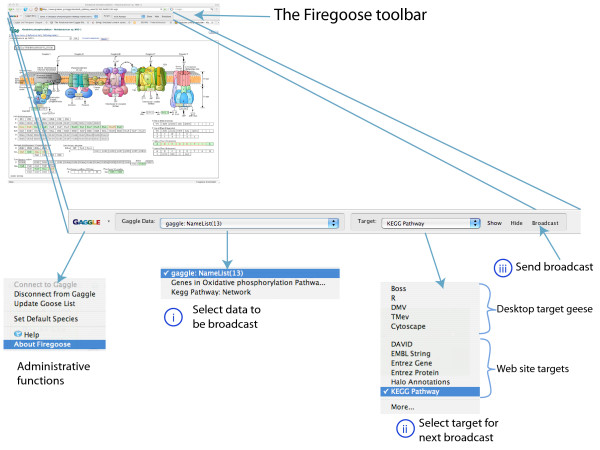
**The Firegoose toolbar for Mozilla Firefox**. A broadcast is sent by three steps: select data to be broadcast (i), select the target of the broadcast (ii), and click 'Broadcast' to send (iii). In this example, a list of genes has been broadcast to KEGG. The result shows that the genes are part of the oxidative phosphorylation pathway.

When data is broadcast to the Firegoose, it can be rebroadcast, under user control, to any supported website. A broadcast to a web resource is typically used to build a query against an underlying database. For example, a set of nodes representing genes may be selected in a Cytoscape network. Broadcasting this data from Cytoscape to the Firegoose causes a list of gene identifiers to appear in the Firegoose's broadcast data menu. To broadcast to the KEGG Pathway database, the user would select the gene list and then select the target "KEGG Pathway". Clicking "Broadcast" submits a request to KEGG for the pathways in which those genes participate (Fig. 2).

More importantly, the Firegoose also brings data back from the web in a usable form. Traditionally, a web server responds to a request by sending an HTML document, which the browser renders for display. The HTML format in which web pages are encoded describes presentation but is poorly suited for representing the underlying structure, or metadata, necessary for integration and re-use by automated processes. In response to this shortcoming, a variety of encodings have been developed including XML, SOAP, and microformats. Firegoose supports several means of data interchange, described in detail below, in order to interoperate with a wide variety of data providers.

#### Screen Scraping

HTML does not represent structured data well. However, by making some assumptions about how the data are formatted, it is possible to reconstruct the structure that is lost in HTML. This process, known as screen scraping, has a long history on the web.

The Firegoose scrapes pages generated by the KEGG Pathways database to acquire a list of genes present in a given pathway. The pathway diagrams generated by KEGG contain links from each gene to its sequence and annotations. The links take the following form: *href="/dbget-bin/www_bget?eco+b0728+b0729"*.

This allows a simple script to glean the information that the two *Escherichia coli *genes b0728 and b0729 play a role in the current pathway, in this case the citrate cycle. By scanning all such links, a list can be compiled of all genes that are members of the pathway in a selected organism, which fits nicely into the name-list data type of the Gaggle. A biologist may, for example, broadcast this list to a visualization tool for microarray data and check for evidence of differential expression under specific conditions of genes whose products play a role in the same metabolic pathway.

While it can be effective, screen scraping is inelegant and prone to breakage. It requires code to be written that is specific for an individual site and will likely require maintenance whenever the site makes formatting changes. For the Firegoose, screen scraping is one means of acquiring data, but is not the preferred option. One simple solution to the lack of structure inherent in web pages is found in microformats.

#### Microformats and Embedded XML

Microformats embed machine-readable data within valid HTML. The structure missing in HTML is supplied in CSS class attributes, giving the parser the necessary clues to extract data systematically from the page. The same data elements may do double duty accommodating both on-screen display and machine-readability. A browser extension like the Firegoose is ideally positioned to augment web browsing with new capabilities enabled by data embedded in the web page.

**Figure 3:**
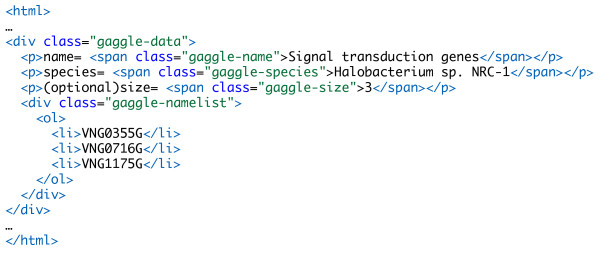
**Example of the Gaggle Microformat**. Microformats embed structured data in web pages by using CSS tags as markup.

We have defined a microformat to represent the Gaggle data types, specifications for which can be found in the Gaggle microformat reference [19]. This format allows any of the Gaggle data types to be embedded directly into a web page, either displayed in the page or hidden from the user. The toolbar detects embedded data and makes that data available for broadcast to other web sites or desktop applications. An example of the Gaggle microformat is shown (Fig. 3) which encodes a list of three genes involved in signal transduction.

As with microformats, XML can be embedded directly in web pages. XML has the advantages of a well-defined syntax and good support by parsers and tools. Adoption of this technique has been hindered by the fact that web pages containing embedded XML fail HTML and XHTML validation. This may not be an obstacle in practice since browsers do not perform validation and simply ignore unrecognized tags. We implemented an XML format for Gaggle name lists to communicate lists of genes from a web view of a database of gene annotations for *H. salinarum NRC-1 *[20].

The benefits of these two approaches are the same. Their value is that they allow data to be captured without requiring site-specific screen scraping code to be written and maintained. By adding a small amount of standard markup to their web pages, website providers make their site accessible to any tools that recognize that format. In the case of the Gaggle microformat, websites can be gaggled without additional code in the Firegoose and a very modest effort on the server side.

Embedding data directly in HTML works well for moderate amounts of data. Where the size of the data is prohibitive, a microformat embedded in the page could provide the parameters needed by tools like Firegoose to access related data through external channels such as web services or direct downloads.

#### Web Services

A web service is a programmatic interface allowing applications running on heterogeneous platforms to interoperate over a network. Typically, the messages passed back and forth are XML. The Firegoose can access web services from within the browser forging a direct link from the presentation of a web site to its underlying data.

For example, KEGG offers extensive web services. The list of genes in a pathway can be requested by calling the *get_genes_by_pathway *SOAP method, which returns the same list of genes acquired above by screen scraping, but in an easily processed XML format. A parameter specifies the pathway and organism in which we are interested. For example, the string "path:eco00020" denotes the tricarboxylic acid (TCA) cycle pathway in *E. coli*. In order to find these parameters, a nominal amount of screen scraping and case-specific scripting is still necessary.

Accessing web services from within the browser is not typical, but offers some compelling advantages. The familiar user-friendly interface of a web application can be used to navigate through a database and then the web service can be invoked to acquire the desired records in structured form amenable to further computation. The browser supports an interactive style of usage without the need to build a customized client to the web service.

Currently, there is no standard way of linking data displayed in a web page with a query to a web service that will return the same data in a structured form. The Firegoose uses screen scraping to fill in this gap. A standardized microformat for this purpose could facilitate this kind of interactive access to structured data.

### Website Handlers

A website may provide data via a web service, an embedded microformat, or other channels. If not, screen scraping may be required. For the sake of modularity, we encapsulate the details of interacting with each individual site or specific format into a separate component called a "handler".

**Figure 4:**
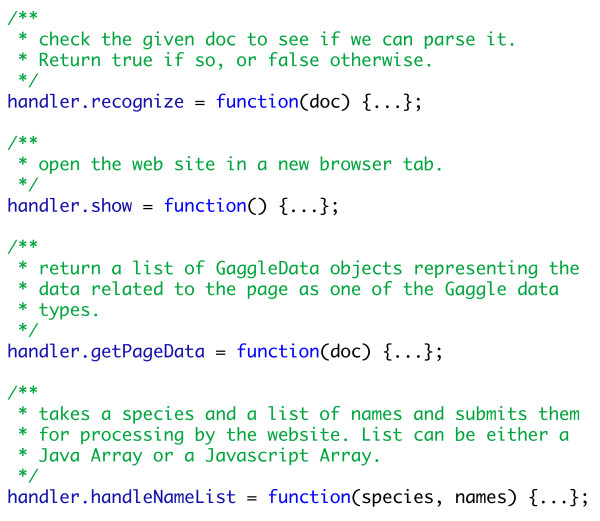
**Interface of a handler script**. The handler interface provides an extension point for adding support for new websites, web services, and protocols.

For each target website or format, a handler is written and packaged in a separate Javascript source file that contains code specific to that target. The handler implements a common interface (Fig. 4) and is responsible for recognizing web pages with which it can interact and transferring data to and from its target. Recognition can be based either on the URL or the contents of the page, which means we can write a handler for a particular web site or a handler that understands a data format embedded in pages from several sources.

When a document is loaded in the browser, the toolbar calls the *recognize *method for each handler until a handler recognizes the document. Then the getPageData method is called for that handler. The handler then inspects the document and constructs a list of GaggleData objects that can be acquired from the page. This, in turn, is used to populate the broadcast menu.

The GaggleData object represents data from the website in the form of one of the Gaggle data types. Support for lazy instantiation and asynchronous access to web services can be neatly hidden behind its simple interface. Typically, getPageData performs only enough work to generate descriptive information for each data object. The actual parsing or issuing a request to a web service can be deferred until the user requests a broadcast. This minimizes processing overhead and prevents unnecessary network traffic.

Any of the Gaggle data types may be returned from the getPageData method, but so far we have only implemented broadcasting lists of names to web resources. Typically, a broadcast to a website is transformed into a query, which retrieves information relevant to a list of genes, proteins, or other identifiers. In principle, methods could easily be defined for each of the other data types: handleNetwork, handleMatrix, etc. In keeping with the nature of dynamic languages like Javascript, all of the "handle..." methods are optional.

## Results and discussion

### Case Study: synthesis of a model of anaerobic physiology in H. salinarum NRC-1 using Firegoose

To demonstrate the effectiveness of the Firegoose in a typical systems biology type of investigation, we explore expression in *Halobacterium salinarum NRC-1 *in response to fluctuating oxygen concentration [21]. Briefly, *H. salinarum NRC-1 *is a halophilic archaeon with a small genome of 2.6 Mb that encodes ~2,400 protein-coding genes [22]. This organism is most prolific in aerobic conditions but switches facultatively to other modes of energy production in anoxic environments. We will take as our starting point a list of genes that were found in microarray experiments to be actively expressed under low oxygen conditions. To understand the physiological changes associated with the anoxic state as completely as possible, we need to understand the functions of individual proteins, metabolic pathways encoded by genes of known function, and functional associations among genes through evolutionary and literature analysis. Not all of this information is contained within one resource; for instance whereas KEGG specializes in information regarding metabolic pathways, STRING calculates functional associations among proteins through comparative analysis of sequence, literature and publicly available experimental data, and DAVID classifies proteins into enriched functional clusters. Finally, using our own expertise we have curated function assignments to many proteins in *H. salinarum NRC-1 *using a combination of sequence and structure-based approaches. In this example, we will demonstrate how the Firegoose can significantly aid in functionally characterizing a set of genes by enabling seamless exploration and integration of several web-based tools from separate providers including KEGG, EMBL STRING, DAVID, and an in-house annotation database for *H. salinarum NRC-1*. First, we will briefly explain how these genes were identified.

Halobacterial cells were subjected to differing levels of oxygen and samples were collected at varying oxygen concentrations and time. Total RNA from these samples was analyzed using microarray analysis [23]. The DMV (Data Matrix Viewer) and the R statistical package were used to normalize and select genes whose expression was significantly changed in response to the perturbations in oxygen. MeV, a microarray data analysis tool, was then used to cluster the differentially expressed genes by their expression profiles. Two sets of genes emerged, one activated in the presence of oxygen and another activated in absence of oxygen. Additional details about this part of the analysis are included in the tutorial web page [24]. We will concentrate on the anaerobically induced genes.

**Figure 5:**
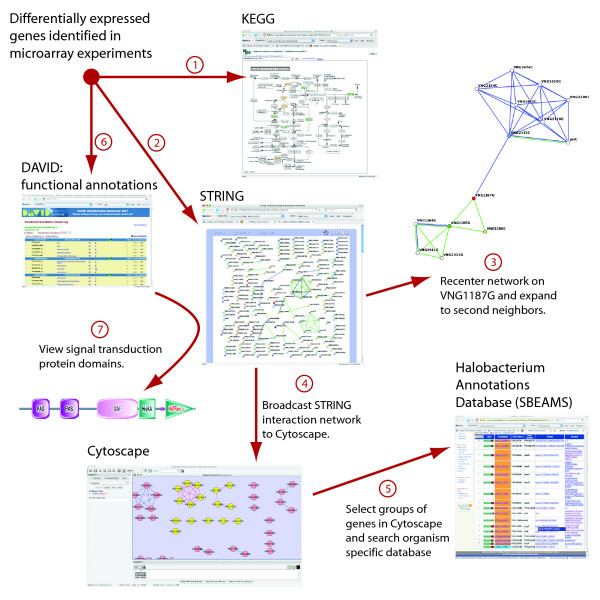
**Using Firegoose to investigate transcription response of *H. salinarum *to anoxic conditions**. A list of genes with similar expression profiles is found by microarray analysis. (1) We broadcast these genes to KEGG to query for known biochemical pathways, for example, viewing the Arginine and proline metabolism pathway. (2) We broadcast the genes to EMBL STRING, a protein interaction database, where we can (3) navigate to functionally related genes. (4) In order to manipulate the network, we broadcast it to Cytoscape. (5) A local database provides additional information. (6) DAVID performs functional clustering. (7) For one cluster containing signal transduction genes, we use String's links to protein domains.

The remainder of the case study traces our inquiry into the functional roles these genes may be playing. Using Firegoose, we consult multiple remote data sources and transfer data between them and local desktop tools. For demonstration purposes, the lists of genes in both aerobic and anaerobic clusters were encoded in the Gaggle microformat and embedded in the tutorial [24], which documents the steps of the analysis. The reader can install the Firegoose, browse to the tutorial web page, and easily reproduce the analysis that follows and is encouraged to do so. The analysis is broken into numbered steps to correspond with data transfers labeled in red in the diagram (Fig. 5).

#### Step 1

We first consult KEGG, a database of curated biochemical pathways. Starting at the tutorial web page, we broadcast the embedded anaerobic gene list to the KEGG Pathway target. This has the same result as cutting and pasting into the KEGG interface; KEGG performs a query for biochemical pathways in which these genes participate. Thirty-one of the 222 genes that were queried matched KEGG pathways including amino acid metabolism, ABC transporters, and active potassium transport. Some of these pathway matches are consistent with known physiological properties of *H. salinarum NRC-1 *such as to facultatively derive energy by fermenting arginine under anaerobic conditions. Further, this analysis also suggests that uptake systems for several alternate nutritional sources (e.g. glycerol) may also be utilized under anaerobic conditions. Interestingly, KEGG also finds two transcription factors in this anaerobic set, *tfbA *and *tfbE*, providing clues to possible regulatory mechanisms.

Although the information gathered from this first line of analysis has yielded considerable insight into the physiological adjustment to an anoxic environment, 191 genes did not match any enzymes within the KEGG catalog of pathways. At this point, we could narrow our investigation to genes in a particular pathway or to the unmatched genes, which Firegoose can capture and broadcast onward. But, for the next step, we'll stick with the full set of anaerobically induced genes.

#### Step 2

To continue our analysis, we use EMBL STRING, a powerful tool focused on protein interactions computed from supporting evidence such as sequence homology, journal abstracts, and protein domains. Specifically, we are interested in finding functional associations among genes already classified into metabolic pathways by KEGG and genes of unknown function. We broadcast our list of anaerobically induced genes from the tutorial page to STRING, which responds by displaying a network of nodes (proteins) connected by colored edges representing functional relationships.

We find strongly interconnected components of the network that are associated with specialized metabolism and do not have corresponding pathways in KEGG. For instance, a cluster of six genes appears in the network connected by edges indicating chromosomal proximity, co-occurrence across genomes, and text mining results. STRING gives annotations and protein domains that show these genes to be involved in the use of dimethyl sulfoxide (DMSO) as an alternative electron acceptor [25]. A second gene cluster, connected through chromosomal proximity associations, encodes gas vesicles used by the organism to vertically orient itself in the water column [26].

#### Step 3

Another interesting grouping links four genes of unknown function *VNG1183H*, *VNG1184G*, *VNG1185G*, and *VNG1187G*. VNG1187G contains two multicopper oxidase domains. VNG1185G is annotated as a Coenzyme PQQ synthesis protein and VNG1184G is annotated as a heme biosynthesis protein. Recentering the network on VNG1187G and expanding twice (to reveal second neighbors in the network) shows functional relationships to several other proteins including more heme biosynthesis proteins. Because not all of these genes were differentially regulated in oxygen the initial seed generated from expression analysis may have captured some incomplete pathways including this particular one. However, by combining, filtering and expanding that seed using a variety of resources we can navigate towards a comprehensive picture.

#### Step 4

STRING makes its networks available in an XML format, which the Firegoose can parse and broadcast to other Gaggle tools. Broadcasting the network to Cytoscape allows the user to work with the network more interactively. For example, subsets of nodes can be selected and broadcast, or highlighted in response to broadcasts from other tools. We select the four-gene cluster noted above and broadcast to the Firegoose for further investigation.

#### Step 5

The *H. salinarum NRC-1 *proteome has been recently re-annotated using a combination of sequence and structure-based approaches. These functional annotations are curated and made publicly available on the web [27] through SBEAMS[20], an open source data management system. In this database, VNG1187G is annotated as a putative copper containing nitrite reductase. Drilling down using the links provided within the SBEAMS database we see that the supporting evidence is a match to entry *1KBV *(the membrane protein AniA, a copper-containing nitrite reductase from *Neisseria Gonorrhoeae*; e-value < 10^-166^) in the Protein Data Bank. This function in conjunction with the functional associations with putative heme biosynthesis proteins and a putative coenzyme PQQ synthesis protein form the basis of a hypothesis that *H. salinarum NRC-1 *may possess a nitrite reducing pathway.

#### Step 6

DAVID is a functional annotation tool that integrates many of the same primary sources as KEGG and STRING but displays its results in tabular format rather than as a network.

DAVID differs from the other resources we have used in that it doesn't work well with the VNG naming system used to identify genes in *H. salinarum NRC-1*. We use a Gaggle integrated translator utility to translate gene identifiers between the VNG nomenclature and GI accession numbers suitable to be broadcast to DAVID. The issue of multiple naming schemes is a major bottleneck for data integration in biology. Passing broadcasts through a simple synonym-mapping translator within the Gaggle helps overcome this hurdle.

DAVID also clusters genes with related annotations, conveniently summarizing the kinds of cellular processes for which a set of genes is enriched. Our genes of interest divide into 17 clusters — several of which were consistent with the results from previous steps. For example, seven genes, including one member of the DMSO cluster, have products annotated as being involved in electron transport.

#### Step 7

DAVID produces another cluster containing signal transduction proteins. Back in STRING, we can click on these proteins to view functional domains provided by the SMART database [28,29]. VNG0355G (Htr14) contains sensory domains associated with chemotaxis. Both VNG0716G (AfsQ2) and VNG1175G (PhoR) contain domains implicated in signal transduction and light sensitivity. It is notable that this is biologically meaningful because light and oxygen physiology in *H. salinarum NRC-1 *are tightly coupled with one another and also with physical relocation (taxis).

### Summary of results

In this example we have combined four web-based resources with several desktop applications to build a unified understanding of the physiology of *H. salinarum NRC-1 *under anaerobic conditions. The depletion of oxygen seems to activate certain aspects of amino acid metabolism, and alternate energy transduction pathways such as phototrophy, arginine fermentation and DMSO respiration. The simultaneous induction of gas vesicle synthesis is consistent with the anaerobic physiological behavior of *H. salinarum NRC-1 *to move towards the surface in search of alternate energy sources including light. The data also suggest that *H. salinarum NRC-1 *may have some specific nutritional requirements in this anoxic environment that cause the induction of an array of membrane transport systems and possibly previously uncharacterized functions such as dissimilatory nitrite reduction. In addition, putative components of the signaling and regulatory mechanisms that may mediate the transition to this metabolic state were also detected.

**Figure 6:**
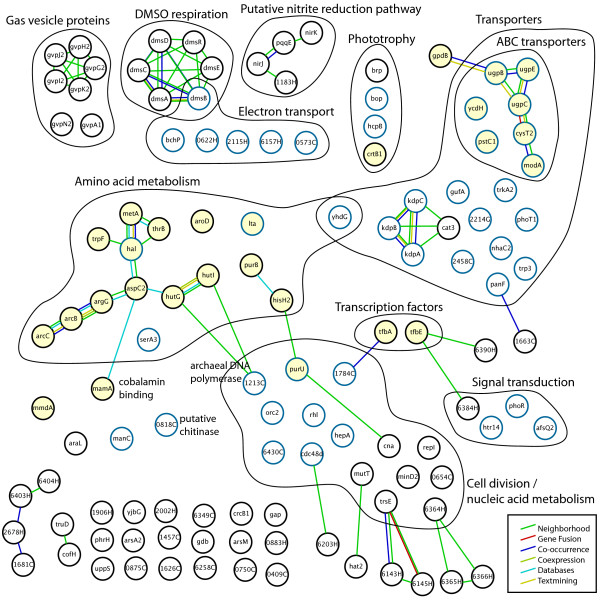
**Integrated visualization of *H. salinarum *response to anoxic conditions**. The transcriptional response to anoxia was characterized using several data sources. Edges represent several types of evidence for functional association provided by STRING. Yellow filled nodes indicate genes classified by KEGG. Blue outline nodes indicate genes classified by DAVID. Other nodes were characterized by an in-house annotation database or other sources, including PFAM, BLAST, and PDB. 102 genes of unknown function were omitted.

It is important to recognize that the information resources, while overlapping to some degree, provide complementary perspectives. KEGG provides information regarding biochemical pathways but does not draw associations between these pathways and genes of unknown function. This information can be obtained from STRING, which on its own is not cognizant of the metabolic pathways. Likewise, DAVID includes information on functional domains within proteins and integrates many of the same primary sources as KEGG and STRING. But, DAVID presents the information differently with its unique clustering feature to provide a statistical evaluation of the enrichment of particular functions among the queried genes. Finally, our local database provides a source of data curated manually by experts through years of careful literature surveys and experimentation. Integration of these web-based resources with desktop applications through the Gaggle and the Firegoose enables the type of analysis that is necessary to understand the complex dynamic regulation of cellular responses (Fig. 6). Complete data from each of our four sources can be found in the supplementary table (Additional file 1).

### Comparison with workflow software

Firegoose, along with the Gaggle framework, shares several features in common with workflow tools such as Taverna[30,31]. They share the strategy of composing distinct programs and data sources to build larger systems with rich capabilities. To this end, both Firegoose and workflow tools benefit greatly from the availability of programmatic access to structured data and computational services over common web protocols.

Workflow tools enable a user to automate a well-defined and repeatable process, often using web services or message queues for interprocess communication. But, before a well-defined analysis process exists, there is a need for exploratory analysis, which is necessarily ad-hoc. Gaggle and the Firegoose seek to enable this kind of interactive exploration by exploiting the flexibility of web-based tools in combination with desktop analysis and visualization tools. Scripting an interaction with a web site is, of course, possible using a browser extension, but the emphasis in the Firegoose is on automating the exchange of data, leaving the direction of the analysis up to the user. The difference in emphasis does not rule out using workflow engines and Firegoose together. Invoking workflows on a remote server from within Firegoose is one potentially valuable example.

### Future Work

Additional functionality could be added to the Firegoose in a number of ways, most easily through the addition of more handlers for biological websites. We also considered that users might want to develop their own handler scripts. We prototyped code for dynamically importing custom scripts into the Firegoose. Other projects such as Greasemonkey [8] have had success with similar capabilities. If further developed, this feature would allow a straightforward mechanism for users or data providers to contribute scripts.

Supporting the RMI communications protocol requires Java. Using Java within a Firefox extension is something of a challenge and code from MIT's Simile project [9] was extremely helpful in this area. An alternative under consideration is to communicate with the Boss using an XML based protocol over sockets eliminating the need to run a Java virtual machine in the browser's process.

We plan to extend the Gaggle microformat to express links to data in addition to embedding data directly in the page. This allows large data structures to be transmitted independently of the page while preserving the linkage between presentation in the browser and the underlying structured data. A standard format would decrease the need for customized coding for each web site.

RDF (Resource Description Framework) is a data model designed to represent meta-data for Semantic Web applications. Incorporating support for RDF into the Firegoose would allow the Gaggle to exchange data with the semantically rich resources envisioned by proponents of the Semantic Web project.

## Conclusion

The Firegoose incorporates Mozilla Firefox into the Gaggle environment providing coordinated access to web applications and programmatic data sources. Performing data integration in the browser has several advantages and is perhaps the most interesting feature of the Firegoose. Browsers excel at search and navigation. Using the Firegoose, a biologist can search and navigate web resources using familiar browser-based interfaces with the additional capability of easily moving data from one web-based resource to another as well as between the web and the desktop. Interactively integrating specific information as needed replaces the cumbersome process of maintaining local copies of large databases and manually coercing data from diverse sources into a compatible format. Using the Gaggle data types as intermediaries lowers the barrier between web resources and desktop tools, allowing the scientist to creatively combine and re-use data in ways that go beyond those provided by the curators of individual data sources.

The Firegoose positions the Gaggle to take advantage of increasing use of web protocols to transmit structured data. The Firegoose provides a framework in which new web resources can be integrated into the Gaggle in a straightforward and easily implemented manner, accommodating a variety of protocols. In supporting a number of protocols, we hope to encourage data providers to make available structured data in the format of their choice and to provide the necessary information to link web interfaces with the underlying data allowing browsing and programmatic access to become seamlessly integrated.

If the web is becoming a channel for structured data, applications that share data between diverse web resources and software tools will be of increasing importance. The Firegoose aims to fill this role for the systems biology domain.

## Availability and requirements

Source code for the Firegoose, along with that of the other components of the Gaggle, is available at the Gaggle website [32]. Also available are instructions for installing and uninstalling the Firegoose toolbar [33] and documentation [34]. Most of the desktop components of the Gaggle are deployed as Java webstarts, which can be launched by clicking a link in the browser.

The toolbar is compatible with versions 1.5.x and 2.0.x of Mozilla Firefox. We anticipate maintaining compatibility with Firefox 3.x when released.

Java version 5 [35] or higher runtime environment is required and the Java browser plug in for Firefox must be installed. Extra attention is often required to install the Java browser plug-in on Linux. Specific instructions for most distributions are available on the web.

The source code is distributed under the GNU Lesser General Public License, the text of which is available at: .

## Authors' contributions

JCB Wrote the manuscript and implemented software.

PTS Conceived and initiated the project, implemented prototype and provided feedback on the written manuscript.

AKS Assisted in the conception and implementation of the case study and provided feedback on the written manuscript.

NSB Conceived and initiated the project, wrote the manuscript and provided direction and feedback on quality of results and software design.

All authors read and approved the final manuscript.

## Supplementary Material

Additional file 1 **Characterization of *H. salinarum *anoxic transcription response by four data sources**. Data about our genes of interest from KEGG, STRING, DAVID, and the Halobacterium genome annotations database are presented here in tabular form. Click here for file
